# Developing principles of social change as a result of a Pasifika Youth Empowerment Program: A qualitative study

**DOI:** 10.1002/hpja.395

**Published:** 2020-08-19

**Authors:** Ridvan Firestone, Anna Matheson, Justice Firestone, Max Schleser, Emily Yee, Hana Tuisano, Keawe‘aimoku Kaholokula, Lis Ellison‐Loschmann

**Affiliations:** ^1^ Centre for Public Health Research Massey University Wellington New Zealand; ^2^ School of Health Faculty of Health Victoria University of Wellington Wellington New Zealand; ^3^ Department of Film and Animation School of Arts Social Sciences and Humanities Faculty of Health, Arts and Design Swinburne University of Technology Melbourne Australia; ^4^ Department of Native Hawaiian Health John A. Burns School of Medicine University of Hawaii at Manoa Honolulu HI USA

**Keywords:** adolescents, capacity building, community development, community health, empowerment, health equity, obesity, Pacific health, youth

## Abstract

**Issue:**

Empowerment is a concept over‐used in health promotion, yet it is an important process that can used in developing the capacity and capability of young people for creating social change to improve healthier lives.

**Methods:**

The Youth Empowerment Program (YEP), a pilot study aimed at empowering 15 youth (18‐24 years) to lead healthier lives. We present secondary outcomes of the original YEP study, using focus groups and mobile‐mentary approaches to capture the impact of the YEP through the youths’ understanding of the program. Thematic analyses to examine the pragmatic usefulness of the empowerment program.

**Results:**

We identified three major themes: (aa) *Knowledge*: education and awareness of healthy living and understanding of the wider social health issues, compound the health complexities of obesity; (b) *Youth as catalysts for change*: the youth viewed themselves as agents of social change; and (c) *Transformation:* the youth recognised themselves as catalysts for change that can positively transform communities into action.

**Conclusion:**

This study contributes new insights and depth of understanding about how the empowerment program can strengthen the process of individual capacity in an effort to mobilise social change for the betterment of the whole community, particularly among indigenous Pasifika population groups.

**So what?:**

Developing empowerment principles will enable others to consider “how apply” empowerment more practically when working with young people and not use it flippantly with no real action‐oriented outcome.

## 1 INTRODUCTION

New Zealand (NZ) has a large proportion of Pacific peoples residing here, with over 8% of the total population self‐identifying as being Pacific and Samoans make up almost half of the total Pacific population.^1^ Regarding health, NZ has one of the highest rates of obesity internationally.^2^ Nationally, obesity disproportionately affects Pacific peoples (67%), compared to the general population (31.3%).^3^ There are significant health and social inequities in accessing care and outcomes for Pasifika peoples.^4^ Community‐based participatory research has enormous potential to draw on the innovative potential of Pasifika youth, their knowledge, resources and motivation to address their experienced inequities. A novel approach to obesity prevention would be through tailored empowerment programs for youth. Previous work has utilised youth as agents of change as part of a social movement, which have shown to be powerful approach if given the adequate resources.^5^, ^6^, ^7^ Such programs are needed for health promotion programs and it is a good fit with Pasifika peoples—a valid approach to empower youth in the efforts to reduce health inequities and reshape community health.

There have been several community‐based participatory studies^8^, ^9^, ^10^, ^11^, ^12^, ^13^, ^14^ that highlight the successes of working with population groups to tackle obesity and other related NCDs (eg, T2DM) including the effectiveness of culturally adapted intervention approaches to manage obesity health‐related diseases.^15^ A similar study by Townsend et al (2016) described the community‐based participatory research approach (CBPR) and data collection process that incorporated active decision making and leadership by community partners, and from the participants themselves. CBPR was considered a positive research approach to establish trust and integrate community expertise, particularly when it comes to integrating and understanding other scientific components such as behavioural and biological sciences.^16^ A movement involving an ecological social‐change approach^17^ including key partners within a community setting such as youth, other community constituents (eg, staff and agencies) and relevant sector (health and non‐health) stakeholders may offer another way forward in terms of addressing NCDs.

There are many youth empowerment programs available, however, more often than not, these programs tend to be located or carried out within a school setting, with a focus on “teaching” the young adolescents.^18^, ^19^, ^20^ Empowerment programs should be more than extending providing new knowledge and understanding, it should also provide a social space for young people to transform knowledge into action; identify and learn to overcome barriers; and develop leadership and confidence in social action activities.^21^ This should be the crux of empowerment programs. Building “capacity” to improve health equity and tackling the social‐health disparities experienced by, often, marginalised communities, is becoming increasingly necessary. Addressing the needs of a community by investigating the health issues through the perspectives and lived experiences of the community and its constituents using a bottom‐up approach requires time, effort, resources and funding.^22^ Mobilising the community into collective action is viewed as a pragmatic re‐distribution of resources and it is an indicator of community empowerment.^23^, ^24^

The over‐use of the term empowerment can lead to a diminished meaning, mainly because the term has been borrowed from many disciplines, whilst others confuse “power” with “empowerment.”^24^ From a health promotion perspective, empowerment is the “process of enabling people to increase control over, and to improve their health.”^25^ Historically, empowerment has shown to be rooted in the civil right and women’s movement, derived from social action ideology in the 1960s.^26^ Furthermore, when individuals living in oppressed societies, re‐construct their personal and social realities, this can lead to citizen empowerment, where they become assertive and committed grassroots activists.^26^ Based on the literature, personal development, participation, raising social consciousness and social action are the key features of empowerment programs.^18^, ^19^, ^21^, ^26^

This paper aims to describe through the youth empowerment,^21^ the perspectives of youth, their learned view of the empowerment process and the practical implications of the program, particularly as community members who are capable of taking ownership of obesity as a complex health problem affecting Pasifika peoples.

## 2 METHODS

At the completion of the YEP program, we conducted: (a) two focus groups (n=15 youth, per group) and; (b) piloted the use of individual mobile‐mentaries^27^ (with the same 15 youth), as a form of digital narrative to further understand the impact of the YEP at an individual level, using Pacific talanoa^28^ methodology. Talanoa as a sharing concept is widely accepted by different Pacific ethnicities. The facilitator ensures the environment is welcoming and respectful. The participants are open to share their perspectives and ideas, and this is often based on a trusting relationship. The key questions used in the focus groups and mobile‐mentaries are listed in Table 1. The data from the focus groups and individual mobile‐mentaries were used for this paper, to triangulate the knowledge obtained at a group (focus groups) and individual (mobile‐mentaries) levels. The aim of the focus groups and digital narratives were to capture the impact of the YEP through the youths’ understanding of the program. The empowerment approach enabled youth to investigate the social world through their lived experiences and perspectives. Thus, for this study, we used a CBPR approach aligned with Pasifika methodological processes to understand the impact of the YEP program through the perspective of Pasifika youth.

**TABLE 1:** Questions for focus groups and mobile‐mentaries

Key questions	
Focus group 1	Mobile‐mentaries
How as the YEP changed you and why is it important to you?	How did you find the YEP program?
What were your inspirations from the YEP?	How has it impacted you?
What were your fears from the YEP?	What was your highlight?
What do you perceive as your strengths?	What did you find challenging?
What do you perceive as your passions?	What did you expect to gain from it?
What are the most common stereotypes?	Favourite module and why?
Focus group 2	How are you going to apply and implement what you’ve learnt from YEP in your life?
After hearing about the key themes developed from our first meeting, how do you relate these themes to the pillars of social change?	How did you find the action planning process?
What are the ways in which we can inform other youth or groups who may go through this same or similar program?	What does the intervention mean to you?
What cultural and social norms do we need to consider when we are thinking about building or running a similar program like the YEP?	What do you hope to achieve with the intervention?
	What are the challenges and how will you overcome them?
	What’s the ultimate goal for the intervention?
	How will you drive the intervention message? Raise awareness?
	How are you going to drive this change?

The methodology and initial findings for the current project has been published earlier,^21^ where we described the overall study context including the empowerment process. In brief the sampling strategy involved recruitment of 15 youth aged 16‐24 years. All participants had self‐identified as being Pasifika. The main ethnic groups represented were Samoan, Tongan, Cook Islander, Fijian and Tokelauan. This age group was targeted as: (a) 21% of this age group are reportedly obese and this rate is projected to increase steadily^29^;(b) it is a “youthful population” and during these formative years they are open to change, particularly in developing health promoting behaviours and lifestyles^30^; (c) they are also typically at a time in their lives when establishing their place and identity within a community which is important; and (d) young people understand, have lived‐experiences and often are the targets of social marketing products that promote an obesogenic environment.

Focus groups are a well‐established form of social research, and they were facilitated and digitally recorded by the first named author. Digital narratives have also been used widely in different formats and under different paradigms.^27^ Specifically, using their own smart‐phones, the youth were trained by MS utilising story‐boarding and the set of questions outlined in Table 1 to document their independent narratives about the health‐related issues of obesity. The initial focus group set out to establish how the YEP had an impact on the youths’ knowledge and awareness of obesity and its related health problems. In the second focus group, we identified and utilised the key themes (from the first focus group), as discussion points, to identify aspects of social change, from a youth perspective. The approach aligns with aspects of the theory of social justice leadership approach.^31^ This theoretical approach was deemed appropriate, because the empowerment process sought to identify leadership qualities in young people as a process to express their views of the social justice issues concerning their communities.

The focus groups and individual mobile‐mentaries were transcribed verbatim and entered into an NVivo 11 software program and then, thematically analysed independently by the author and again by an independent research assistant, using a general inductive reasoning approach. This approach allowed for iterative coding, until meaningful thematic categories were attained (saturation), as well as it being determined by the aim of the project and by the participants. The transcripts and a summary of themes were presented to the youth and community leaders as a process of authentication, which aligns with the CBPR and Pasifika methodological processes. No further modifications were made and the participants corroborated that the transcripts and summary reflected a true and accurate record.

## 3 RESULTS

### 3.1 Focus groups

Two major themes were identified from the focus group discussions. The first, being that; *“knowledge is a powerful tool for social change*.” The youth reported that “knowing and understanding” obesity and its related issues was valuable for recognising the consequences of this condition on personal health and the impact on family members. Although the youth still identified with body size and over‐weight as the main reasons for obesity, they also expressed a deeper understanding of the social‐economic and cultural factors that impact health behaviours, recognising that these were less well understood, as often knowledge was “handed‐down” from less informed [older] family members.

“We get stuck in our own familial ways, often handed down by families and cultural ways.”

“YEP is a massive eye‐opener, forces us to think about how to change our foods and re‐educate the family and community.”

Through the program, the youth achieved a clearer understanding that their personal experiences of chronic diseases, such as obesity and diabetes, differ greatly to that of their older family members. The module on the historical factors^21^ of healthy lifestyle in the Pacific over 200 years ago, highlighted the major differences in the way their ancestral elders worked, lived and consumed food (traditional vs the conventional three meals per day), than how their parents are living today. Furthermore, the participants learnt that “*obesity is more than just about health and living.”* One youth’s comment illustrates this point:

“In this generation what we are exposed to is always different, like my great grandparents, their experiences were just eating breadfruit and one chicken for everyone, and then the next generation they have a bit more stuff … and we have all this access to a lot of things and I think that’s why we are always sick of.... like back in the islands we always have live food, bananas then a sua i’a (fish soup). But now it’s like oh we can go to one of the restaurant to get fried rice, there are so many choices and that’s what’s … going to danger yourself.”

From the YEP program, the youth gained an understanding of the inter‐play between the social, political and cultural factors, as highlighted through their “lack of experience and knowledge” of the accessible healthy food option, budgeting and planning for meals. This highlights the essential need for capacity building among youth to better grasp the key factors that may contribute to the issues of obesity for Pasifika peoples. They also learnt that there is a correlation between long‐term conditions (obesity) and poverty. That is, those living in deprivation have limited opportunities and choices, tend to experience greater stress and these factors have the propensity to evolve to high‐risk behaviours.^32^

“Learnt about the political nature of it … A lot of the solutions are palagi (European) focussed… They don’t cater for Pacific Islanders especially programmes to address the health needs of Pacific families that have 10+ members in it. Individually yes its fine but we live as a family/community.”

“… the way I see it, is that [the] Government is just good at pin pointing it but yet they don’t come up with better solutions that’s healthy eating [from a Pasifika perspective]. But that it’s so expensive for a Pacific Island family … like yea it’s cheaper to go and buy a packet of sausages cos a 20 pack of sausages can feed a family of five, than it is to go and buy like 3 chicken breasts ‐ it’s more expensive than sausages.”

Another common thread of explanation, was that obesity is the “cultural norm” for Pasifika peoples, however, the youth gained a better understanding that there are major game‐players (eg, marketing and advertising, cost of cheap food) that influence decision‐making processes in order to cater for families to survive.

"as a culture … when I was growing up it was like eat! You carry that on when you are older, that’s all you know and we can’t just sit there and we judge people who are big… that’s all they know and that’s probably their norm. I’ve got afa‐kasi [half‐caste] cousins in my family, when we want something to eat and we would go over [to their] house and you open their pantry and there would be like half bags of chips open in the pantry with the pegs on it, me and my brother and sisters would laugh. If it was our house we would have eaten it all.”

The second major theme identified was: *“youth can be catalysts for social change*.” The YEP program took a proactive approach to empower Pasifika youth to “investigate to mitigate” social change. It is not in Pasifika culture to ask questions or to make inquiries about issues, especially about health and body size, as this can be seen as being disrespectful. However, the YEP has provided an opportunity for the youth to recognise that they are important resources for their communities.

“YEP taught me about Pacific health and how it affects our community. You can apply the tree analogy to any health issue to help understand or teach people the different views of an issue.”

“Could include the role of families and church in the empowerment process.”

It is clear from these examples that the program encourages the youth to use the power of their expression to help improve the health of their communities. Of note, the “tree analogy” refers to a three‐tiered analytical framework that participants had undertaken as part of the empowerment program to dissect personal views, de‐mystify traditional tales and improve understanding of the core issues of obesity. In short, the analogy of the tree (branches: visible issues of obesity; trunk: systemic issues; and the roots: hidden and deep issues of obesity). This analogy resonated well with the youth and it described in detail elsewhere.^21^

### 3.2 Digital narratives

Transcribed data taken from 13 individual mobile‐mentaries were coded and analysed using thematic analyses. We identified two major themes (a) *Concerns for health in the community* and (b) *social change for healthy transformation*.

For theme one: *Concerns for health in the community*, there were several common links indicating how concerned the youth were for their families and the wider community. These included the importance of social connection and of retaining social and cultural values, even while recognising the role of wider influences.

“One way this program has impacted me is recognising … the deeper levels of how this issue has come about and one of my favourite modules … in this program was the … the Mexican feast that we had. It was an eye‐opener in reminding me about what a feast means. It means coming together, stripping away technology, and really being in the presence amongst people that you love and having conversation around healthy food, all your greens. What I loved about it was that I had real conversation with people in real life, and it reminded me that we need to hold tight to this idea of coming together.”

“One of the issues that are important … is how do our values and our culture intertwine with the issues on a national scale and also how we look on a global scale too. I learnt a lot, not only from our facilitators but also bouncing ideas off all of the other participants … I had a really good time discussing the issues and shared concern for the wellbeing and health of our Pacific people. I think moving forward that the Youth Empowerment program is, can be implemented in any area, whether it’s health or whether its other social issues surrounding inequality … and social determinants of health.”

*Education* about health and obesity was also viewed by individual youth as essential to provide an “informed” decision or perspective on what and how health is perceived in the community. It was also viewed as a *driver for change* and a key factor in *identifying and solving health issues* in the Pasifika community. Some examples from individuals included:

“Education is a significant tool to make a change (for success).”

“Lack of education towards the issue means you cannot make a real change.”

“Pasifika community is the most at risk due to the unhealthy living conditions (e.g. obesity).”

“Youth Empowerment! What do I love about it? … One, Empowerment, it is the leadership … facilitating learning to bring about change and … I love it cause it’s life‐changing, transformation. And I think, for me … I hear their narratives and their real powerful stories about empowering themselves, their community and their families … physical exercise, foods, dietary … when you’re in the Youth Empowerment program, you get empowered by others who lead, and learning about how to facilitate change.”

The *health [status] in the community* was seen as having a large effect on the health of the future generation (eg, the environment in which people were raised‐up in, motivation of the family to live healthily). The narrative emerging from all the youth participants was one of the “health of their community” being low quality and in need of improvement due to the experiences of ongoing health issues or untimely family deaths. The consensus amongst all the youth was that*:* *“the health of Pasifika communities*—*should focus on ‘improving’ the health in the community.”* Additionally, there is a need to have a less deficit focus on obesity and body weight and being more mindful of the social environment. The youth also understood that for their generation, *“living healthy means living longer.”* Interestingly, and in line with much emerging literature on the role of social and cultural norms in producing health outcomes,^33^ *culture* was viewed as a pivotal factor in how the community perceived health and obesity issues.

“Pasifika culture views obesity as socially acceptable because it is seen as healthy (skinny bodies are associated with disease, sickness and death etc…).”

“Pasifika peoples’ values and beliefs affects or intertwines with the current health issues revolving around culture.”

Rather than taking the typical Pasifika cultural viewpoint, that; “being big is a sign of good health,” the youth stated that *culture is the difference* between the Pasifika and Westernised viewpoints of health. That is, the cultural values, principles and way of life influence the family and community’s perspective on and practice of health. Neither is views were considered wrongful, just different. However, if we observe the health of Pasifika people through Pasifika‐lens only, it would most certainly be culturally relevant and fundamentally holistic.^34^, ^35^

Healthy transformation (theme 2), also included *“Education”* because exploring the perspectives of obesity‐related health issues and ideating potential causes and solutions to the obesity‐issues were seen as being related to the notion of education (as described above for Knowledge as a tool for social change). *Environmental factors* relating to how key family members were “raised up,” “current living environment,” “health behaviours and habits,” and how the “community and culture” shaped views and practices of healthy living, were all domains of whether individuals, families and communities are open to transformation. Some of the youth excerpts highlight these points:

“We’re stereotyped as … you know diabetes or diabetic people and … we’re seen as, the big guys [like myself]. I’ve learnt a lot too, you know, to put a stop to this … and to create a solution – that’s what we called it in one session, that we are seen as, and known as, a solution in our time … providing help for ourselves and our families, and our community.”

“… just empowering young people to strive to live longer, strive to live better, live healthy, and also the fact that we’re empowered to make some of those changes in our communities, which I think is a really good starting point.”

Also, understanding the role of Pasifika leadership and its significance in how the community relates and interacts (inspires and motivates others) proved a strong common theme among the YEP youth:

“Empower yourself and others to make a change.”

“I think some of the challenges were a bit, trying to apply it to my own life … you can learn all this stuff but it doesn’t mean anything until you put it into action yourself, and you can’t go and empower other people … especially with our program going with the church and with the youth … there’s no point teaching them if I haven’t applied it to my own life.”

One youth was exemplary in illustrating the leadership qualities that can have an impact on enabling community transformation:

… I really hope to achieve positive change however it may be measured I just hope that it brings about awareness to people, that people start taking it seriously that they see how it’s affecting not only them but, the generation to come.

The message that we are hoping to deliver is for anyone and everyone, but mainly our Pacific community. This is where the problem is like crazy, and so we really wanna target the at‐risk community, which is our Pacific people.”

The overall common goal for this program was for the participants to recognise that youth leadership is necessary for the younger and older generational cohorts and an important barrier was overcoming stereotypes and changing or improving their lifestyle habits. Participants also acknowledged that culture, religious and traditional beliefs and values may impede the change needed to integrate healthy habits into life.

## 4 DISCUSSION

Our study has shown that youth, when given the opportunity to consult and learn about chronic health conditions, such as obesity and develop the necessary knowledge and understanding of the disease, they can become important resources for the community, to lead healthier lives. Very few studies take the time to investigate “how” to address the obesity‐related issues, particularly from a youth perspective and in intervention implementation. This study contributes new insights and depth of understanding about how the empowerment program can strengthen the process of individual capacity in an effort to mobilise social change for the betterment of the whole community, particularly among indigenous Pasifika population groups.

Given the focus on the NZ Pasifika population, who have the highest rate of obesity in NZ,^36^ arguably the youth participants in this study have also had the highest exposure to the obesogenic environment.^37^ Therefore, it is reasonable that their perspectives and understanding of the disease will differ from that of academics who have struggled to reduce the health inequalities associated with this growing epidemic, in Pasifika peoples. Furthermore, the youth perspective tends to overlook being marginalised, or do not consider themselves to be a socially excluded group, rather, their narratives strongly evoke empowerment, advocacy, collective promotion and activism to respond to the growing needs of their community.

The primary aim of this study was to investigate the youth perspective on the obesity condition as experienced through the YEP and to identify key factors of social change, as a result of the program framework. Our analysis highlighted key themes: *Knowledge*—a tool for social change; *Youth catalysts* for change; and *Transformation*—to improve community health. From the youths’ perspective, these major themes were viewed as important factors to instigate social change and influence behaviours, beliefs and values within a Pasifika community context.

### 4.1 Knowledge a tool for social change

The youth from this study perceived education and knowledge as an important motivational factor that can influence healthier lives.^21^ It was clear from the study participants, that the knowledge handed‐down from older family members was due to different environmental exposures (education, lifestyle experiences, family and church environment) and that the indigenous‐related knowledge was not always accurate, particularly that relating to body‐size and health. In the present day, with the abundance of knowledge being shared in different forms (eg, social media platforms), youth have a greater exposure to information, as well as, to the obesogenic context and they are more aware of the diversity of food and how to access food in their local environment.^10^ The YEP combined knowledge and skills as essential components to make informed behavioural changes and mind‐set and this was the single most important theme in the current research. Whilst some studies do not report on how much knowledge and skills are needed to change behaviours, there is often an imbalance between the two and the difference may contribute to unsuccessful obesity intervention.^38^

Other researchers have reported that while having the knowledge is important, it is insufficient to effect behavioural change (fruit and vegetable consumption).^39^ Therefore, there is a discrepancy between advocating for population‐based interventions that aim to target greater overall benefit in risk reduction in many people, than by large reductions in just a few people.^40^ The YEP participants conceptualised that having an influence on empowering individuals and community can have a greater chance of success and sustainability, as the process facilitates participation and it is more relevant to the Pasifika context, because they work collectively as a community, not as individuals.

In addition to knowledge (and skills), building capacity to advocate for healthier lifestyles in communities who live in high levels of deprivation, must be viewed as a health gain. Our finding of building up Youth as catalysts for change is critical when perceived as a process of community capacity development. Who else can mobilise community, address social and public health problems with motivated action, and be open to new knowledge and skill development? In short, the answer is our youth. Other areas of community organisation such as governance, community organisational leadership and management processes are pertinent to ensure health prevention and intervention programs will achieve the proposed work and outcomes. However, few organisations have developed an infrastructure that focuses on the capacity development of youth in their community, as a critical health and social resource that could benefit the community at large.

Capacity development from our youth participants was evident in the way they perceived leadership development, acquiring new and informed knowledge and the need to share this learning by way of educating the community about health at an individual and community level. Previous research^41^, ^42^ have endorsed community empowerment, but only if the community identifies with and understands the actual needs and values of a health program. This study aligns with the Labonte and Laveracks’ capacity development work for: (a) building capacity for program sustainability and (b) capacity‐building relationship.^42^ The YEP theme of “youth as catalysts for change” strongly links with the second point, because it implies the communities relationship is bi‐directional, dynamic in nature and capacity development can address the changing needs of the individuals, groups that make up the community.

Previously, we had described the YEP as a potential public health approach to develop culturally relevant prevention and intervention programs and a means to address social justice within the community space.^21^ Yet the current thematic analyses, clarifies and extends the use of youth empowerment to community empowerment, as identified through the final major finding of Transformation—to improve community health. Which includes a range of other factors, such as the role of culture, leadership,^33^, ^43^ education (see Knowledge above) and other environmental factors directly relating to various community domains (eg, family life).

The role of youth should be considered as an important community resource. They are capable of maintaining a sense of autonomy and self‐determination and easily recognise the value of assistance from other agencies that can enable a program to be sustainable,^21^ this demonstrates that they have shared needs, social networks and the desire to participate in a process of collective action. Laverty (1999) exerts this same notion that, *heterogeneous individuals are able to achieve action through a process that involves personal action, the development of small groups, organisations and networks, in effect the development of community*.^44^

Furthermore, the study participants stated that the health of the community needs to consider specific social issues, such as, culture, deprivation and leadership, which may impact on how various community members perceive community needs. The youths’ understanding of a relationship between obesity and poverty is not a novel finding, however it “adds” to the existing knowledge‐base, that the empowerment process could affect change at a community level, to shift their mind‐set and behaviours to a healthier lifestyle trajectory. Capacity development at the grass‐roots level, as experienced by the participants of this study was essential in working towards reducing health inequities, so that the knowledge that is passed down to the younger generation is well informed, than what is currently practiced. The link with community capacity development and, therefore, transformation fits with the definition of community capacity as a “function of capabilities (individual and community) and socio‐environmental conditions,” and it is inherent as a result of their interactions. It is also a function of the resource opportunities or economic, political and environmental conditions in which people live.^45^ For the youth in this study, the transforming the health of their community links to the first and second key findings, however, it should be Pasifika‐relevant, focused and community‐driven (not from a top‐down approach). The limitations of this study can be evident in the exploratory nature of this work and that a scaled‐up approach of the YEP is necessary to fully comprehend the impact of empowerment.

## 5 CONCLUSIONS

This study has highlighted three key findings that we propose equating as principles that can facilitate social change in behaviours, culture and mind‐set. They are: *Knowledge‐ a tool for social change*; *Youth catalysts for change*; and *Transformation of the community,* by empowering the community. The key findings are inter‐linked and are necessary for community organisations to include as part of any form of capacity development programming. The YEP provides the knowledge capacity development for the youth generation. It also provides a medium for which youth can learn to facilitate ways to address social—health issues through the program opportunities. With youth at the vanguard of collective action for the community, knowledge and education about the key issues of health will be important to Pasifika people to enable better healthier living. The establishment and sustainability of any prevention or intervention program, through network collaboration and capacity development of youth is necessary to ensure that our culture and communities are aligned to healthy living. Youth may be better at identifying different approaches that are fitting for our diverse cultures and communities, which will be important for sustainability and long‐term support at an organisational level.

## AUTHORS CONTRIBUTIONS

Ridvan Firestone led the research work of the YEP. Anna Matheson provided qualitative expertise of the YEP. Justice Firestone was a Pasifika student health Intern and carried out the mobile‐mentary transcription and analyses. Max Schleser, led the smart‐phone narratives with the Pasifika youth. Emily Yee, was a Pasifika student health Intern and carried out the focus group transcription and analyses. Hana Tuisano was a Research Assistant and co‐facilitated the YEP on a weekly basis and contributed to focus group data analyses. Keawe‘aimoku Kaholokula reviewed the preliminary analyses and contributed to the writing of the manuscript. Lis Ellison‐Loschmann assisted with preparations of the analyses and initial and final drafts of the overall manuscript.
